# Decontamination of High-Containment Laboratories for Demolition: A Risk-Based Approach

**DOI:** 10.1089/apb.2021.0033

**Published:** 2022-05-27

**Authors:** Jason Tearle

**Affiliations:** Health, Safety and Biosafety Department, The Pirbright Institute, Pirbright, United Kingdom.

**Keywords:** decontamination, high-containment, laboratories, fumigation, disinfection, risk-based approach

## Abstract

**Introduction:**

Decontamination of redundant laboratories, contained plant space, and support buildings used to handle high consequence animal pathogens (North America BSL-3Ag, UK SAPO4) was required before demolition to mitigate the risk that infectious material was released into the environment.

**Methods:**

Given the age of the buildings and their construction, bespoke qualitative risk-based methods were developed by biorisk personnel, in consultation with specialist contractors where applicable. This approach was to give assurance that suitable decontamination was achievable, sometimes through multilayered approaches to disinfection. Time was considered as a contributing factor in decontamination. Different means of achieving decontamination were employed, and waste management was considered as part of the overall process ensuring a “cradle to grave” approach.

**Summary:**

This article describes the challenges and solutions, faced by a UK facility licensed to work on high hazard pathogens, including foot-and-mouth disease virus and African swine fever virus. The risk-based approach (and methods described herein) may also be applicable to routine decontamination of younger buildings, that is, during renovations or temporary derestriction.

## Introduction

During the design and construction phases of new high-containment facilities, it should be in the forefront of any forward-looking planner or user to ensure there is adequate provision for both operational and end-of-life decontamination. In use, laboratories or animal rooms may require routine decontamination, whether by fumigation, surface disinfection, or a combination of both for reasons including emergency response after a spill, prevention of cross contamination between animal studies, or for temporary externalization for maintenance.

In recent years, there has been investment in many countries to replace aging high-containment facilities, many that were constructed >50 years ago. These buildings will not have been constructed to 21st century containment standards, or their integrity may have degraded over time and, therefore, require the development of bespoke (risk-based) decontamination methods, which may present interesting and novel challenges. Given that foot-and-mouth disease virus (FMDV) is highly infectious, has the potential to cause the greatest economical damage and is moderately stable in the environment, it was considered the worst-case pathogen for release and, therefore, this article places emphasis on FMDV, with supporting information from other viruses as applicable.

Global containment nomenclature differs slightly, in the United Kingdom an Animal Containment Level 4 facility is broadly equivalent to the North American “BSL-3Ag.”

## 21st Century High-Containment Facility Decontamination

Modern high-containment facilities are designed with impervious coatings that may be applied to concrete walls creating an easily cleanable box that can be safely and effectively fumigated. Ductwork carrying contaminated air may be fully welded stainless steel with minimal flanged joints, with provision of ports to facilitate fumigation. Drains shall be constructed from either stainless steel or high-performance plastics to allow full flooding with potentially corrosive disinfectants.

In addition, effluent treatment plants (ETPs) may take this further by having full, and automated, Sterilization (steam) in Place (SIP) capability of vessels and pipework where internal surfaces are exposed to contaminated liquid. Insulation may be either closed cell foam (and removable) where possible, or fully encapsulated (e.g., on effluent kill tanks).

## The Challenges

### Inability to fumigate

For fumigation to be successful the space must be sealable to a level that prevents leakage. The reasons behind this are twofold: first, fumigants by their very nature are hazardous to personnel, therefore preventing exposure of people is critical. Second, fumigant must be retained at validated concentrations and time to ensure its biocidal effect. Older (or repurposed) buildings may not have been designed or constructed to high levels of sealability to achieve this or their physical integrity may have deteriorated throughout their life.

### Fumigant effectiveness

For a fumigant to be fully effective, it must act upon clean nonporous surfaces. High-containment construction methods are continually improving; however, there remain older laboratories with features such as suspended ceilings of porous tiles, wooden doors and frames, and domestic style timber boxing (to “hide” plumbing or other surfaces) thereby making access and exposure to fumigant problematic.

### Long lengths of ventilation ductwork

Best practice in ventilation design of high-containment facilities aims to reduce the length of potentially contaminated ductwork, that is, that between the “space” (e.g., laboratory or animal room) and the High Efficiency Particulate Air (HEPA) filters. This length of ductwork is effectively an extension of the contaminated space and, therefore, requires decontamination before removal or decommissioning.

However, the use of common, or banked, filters can result in long lengths (multiple meters) of ventilation ductwork carrying potentially contaminated air. Studies have shown fumigant is able to penetrate up to 15 m into ventilation ductwork of ∼300 mm in diameter under laboratory fumigation^1^; however, these lengths were exceeded by the ductwork in the buildings presented in this study.

### Long lengths of underground drains

Following the principles outlined earlier, it is equally optimal to locate the high-containment ETP as close to the source of effluent. However, the ETP (that had been constructed later than the other buildings in the facility) was situated ∼100 m distant from the laboratories it served creating a significant length and, therefore, volume of drainage that required decontamination.

### Site with a long history

Laboratories may be located on sites with up to 100 years of activity and although most, if not all, of the original buildings will have been demolished, older buildings may remain at the time of decontamination and demolition. The layout of such sites will be driven by decades of periodic development with buildings of different ages interspersed throughout the campus.

## The Solutions

### Time

Viruses have limited viability in the environment, although there is variation both between virus types and conditions (including temperature and the environmental substrate). A log_10_ (90%) reduction of FMDV has been reported after 11 days at 20°C (summer conditions in the United Kingdom), at relatively neutral pH (FMDV is less stable in extremes of pH), and 18 weeks at 4°C (winter conditions in the United Kingdom).^2^ A review of studies has shown that most of the average survival estimates for FMDV are 3 months or less.^3^ In addition, drying alone on nonporous surfaces (plastic and stainless steel) can result in reduction in excess of 2 log_10_ of virus infectivity for both FMDV and African swine fever virus^4^ and a log_10_ reduction of infectious bronchitis virus.^5^

Taking the published literature into account, it is possible to take a risk-based approach to the likely presence of viable virus present at a given time point after scientific activity has ceased in a building. Indeed, in the European Union restocking of farms after an FMDV outbreak can occur relatively soon: “Restocking should not commence until 21 days after completion of the final disinfection.”^6^ In addition, the foot-and-mouth disease (England) Order (2006) refers to periods between 21 days and 1 year before restocking after disinfection.^7^

Furthermore, under the EuFMD Minimum Standards, it is an acceptable protocol to externalize paperwork (i.e., the removal of paperwork, books, and documents in a usable form) from a contained area after 2 years at 20°C after a suitable and sufficient risk assessment.^8^ This is without any supplementary disinfection of the material, such as UV irradiation.

As an example, taking a theoretical worst-case scenario, if, on the last day of a laboratory being active, an unreported spill of high titer FMDV (i.e., 1 mL at 10^9^/mL) outside a cabinet took place. If the building ventilation remained operational and maintained a temperature of 20°C, applying the more conservative inactivation data aforementioned, it would take ∼100 days (∼3 months) for the virus to lose viability. To give a significant margin for error, continuing postactivity operation of the building Heating, Ventilation, and Air Conditioning (HVAC) for 6 months would provide time to be confident that there has been a significant reduction of any residual virus activity to low risk levels.

At least 2 years had elapsed between the last scientific activity in the buildings and the controlled handover to the demolition contractor. In that time, the decontamination of the Microbiological Safety Cabinets (BSCs), laboratories, and drainage had been performed. All work with infectious virus had been carried out in primary containment. Furthermore, some of the buildings had not been used for work with high hazard pathogens for >10 years, and others not at all (e.g., tissues culture facilities), but were within the restricted area.

### Multiple disinfection stages

As fumigation could not be done safely, or effectively, a risk-based multiple disinfection program was implemented as follows (see Table 1 for disinfectant concentrations):

**Table 1.** Summary of disinfectants used

***Disinfectant***	***Application***
Virkon S (1%)	Laboratory fogging
FAM 30 (1:100)	Laboratory surfaces and equipment disinfection and laboratory (above ground) drainage
Sodium hydroxide (12%)	Below ground drain flooding, ETP pipework and vessel disinfection, and HVAC disinfection
Formaldehyde	BSC fumigation

BSC, Microbiological Safety Cabinets; ETP, effluent treatment plant; HVAC, Heating and Ventilation System.

1. Surface disinfection of floors, work-tops, and the workspace of BSCs: These were considered the areas most likely to be contaminated as BSCs were used to manipulate live virus, and horizontal surfaces would most likely be contaminated if there had been a spill due to direct contact or settling out of aerosols. The disinfectant used was FAM 30 (Evans Vanodine).
2. Formaldehyde fumigation of BSCs: All BSCs were fumigated with formaldehyde (20 mL formalin:20 mL water for a 1200 mm BSC as recommended^9^ at ∼18,000 ppm) with the process validated by placing biological indicators (3M Attest 1294) in the workspace, under the work tray and after the extract HEPA (where possible). All cabinets had a chemical indicator (Browne Formaldehyde Control Indicators, Steris) placed in the workspace during fumigation to further confirm the presence of formaldehyde. Studies have shown that the concentration of formaldehyde to cause a color change exceeds that required to inactivate the surrogate organisms used in the commercial indicators aforementioned to validate the process.^1^
3. Surface cleaning and disinfection by a specialist contractor: All surfaces were cleaned, for example, removal of sticky tape/Blu-Tac from the walls and doors, followed by surface disinfection using the disinfectant FAM 30. This included the walls, floors, large equipment in situ both inside and out (fridges and freezers), and laboratory furniture.
4. Disinfection by “fogging” spaces with Virkon S: One of the potential shortcomings of conventional cold fogging is achieving contact with the undersides of horizontal surfaces, for example, under laboratory benching (personal observations). To achieve better coverage, the spaces were “manually fogged,” that is, an operative wearing Respiratory Protective Equipment carrying the fogger (VectorFog C150+) directed the discharge at all surfaces ensuring total coverage with disinfectant.

Two different broad-acting disinfectants were deliberately chosen and used one after the other, FAM 30 and Virkon S (Lanxess) to give additional assurance to the process. FAM 30 is an acidic iodine containing disinfectant commonly used against a range of viruses, including FMDV. Virkon S is also validated against a broad range of viruses and bacteria with chlorine, hydrogen peroxide, and peroxymonosulfate as its active ingredients. Both disinfectants are listed in the Department for Environment, Food and Rural Affairs (DEFRA, United Kingdom) list of Approved Disinfectants, where such a disinfectant must be used, as suitable against FMDV.^10^ As decontamination in this context was in preparation for demolition, it allowed for “disinfection during destruction,” that is the ability to disassemble or partially destroy equipment or parts of the laboratories to allow disinfectants to reach otherwise inaccessible areas ordinarily exposed only by gaseous fumigation. This approach would not be acceptable for routine decontamination.

### Drain disinfection

Foul waste drainage was divided into two categories: above and below ground. Above ground drains generally consisted of standard plastic laboratory drains from the sinks to the floor slab. These drains were flushed thoroughly with disinfectant (FAM 30 > 10 L/sink) and then the traps were left charged full of disinfectant for at least 1 week. The below ground drains (postfloor slab) were sequentially isolated in sections, fully flooded with sodium hydroxide (NaOH) (∼12% solution) and left to dwell for up to 12 h. During the flooding of the drainage system (and all work with NaOH), operatives wore full chemical suits with hoods to prevent exposure.

In addition, an emergency drench shower was locally available in the event of chemical splashes. NaOH fulfilled two functions in this scenario, first it is a potent biocide. FMDV is inactivated readily at pH >9^11^ and NaOH can inactivate a range of viruses.^12,13^ Second, NaOH (also known as caustic soda) is an effective “drain cleaner” found in many commercially available formulas. It acts by hydrolysis of proteins and saponification of fats breaking down residues and exposing any potential contaminants within a foul sewer system.

In addition, soil vent pipes (SVPs aka “stacks”) were also disinfected with NaOH in situ by spraying, cutting, and removal of sections. This process was carried out at least 2 years after the laboratories ceased to be scientifically operational handling infectious material. Furthermore, SVPs were considered to be of very low risk and it also allowed for local disinfection before removing sections for disposal. Alternative approaches that could be considered include jet washing with disinfectant or in situ fumigation if the SVPs are determined to be clean of gross contaminants after CCTV inspection of the internal pipework. The latter may require removal of access hatches or cutting out sections to allow fumigant to enter.

### Ventilation ductwork

As a result of the inability to fumigate the buildings and, therefore, the ductwork, an alternative means to disinfect the ventilation ductwork was developed. This involved sequentially spraying the internal ductwork in situ with NaOH to a length of ∼1 m into the ductwork, before cutting that section off, laying it on the floor and spraying a second time. This process was then repeated and coverage of each 1-m length of ductwork assessed visually for coverage. During this process, a HEPA-filtered negative pressure unit was installed at the end of the duct to draw the NaOH mist produced away from the operatives.

Applying a liquid disinfectant in this manner allowed penetration into seams and joints in the ductwork. This was of importance as the ventilation ductwork was connected by a variety of methods, including bolted flanges and rivets (note: some of this was contained ductwork within a contained space) and not welded as may be expected in a modern high-containment facility. Prefilters were sprayed with NaOH to wet fix and treat surface dust and then bagged for incineration.

### HEPA filters

HEPA filters from BSCs were fumigated using formaldehyde, removed, and placed into bags before being sent offsite for incineration. Building HEPA filters were not fumigated in situ as the infrastructure within the building was not present to do so safely. Therefore, the filters were removed after the time periods described previously to reduce the risk of viable material being present (>2 years) by operatives wearing Tyvek suits and gloves, and immersed in disinfectant (FAM 30) before being placed in two plastic bags, each zip-tied and incinerated.

One type of HEPA element installed in buildings were drum-type filters (MC Air Filtration). Because these elements filter “inside-out,” it is possible to remove the cap of the housing without exposing the environment to potential contamination. Given that the housings had no decontamination ports to facilitate fumigation, these filters were decontaminated by removing the caps, filling the housing with disinfectant, and leaving to dwell for >7 days to allow the disinfectant to penetrate the filter media before removal.

### Effluent treatment plant

The ETP presented a challenge as all contaminated vessels and pipework needed to be exposed to disinfectant before removal. The decontamination of the building and external surfaces of the process plant was like that used in the laboratories. Detailed examination of the piping and instrumentation diagram (P&ID) was necessary to identify all the pipework, valves, and vessels that could have been potentially contaminated with untreated effluent. Once this process was complete, an operational sequence was developed to ensure NaOH could be pumped around as much of the system as possible. This also identified areas where supplementary disinfection protocols were required.

**Figure 1.**
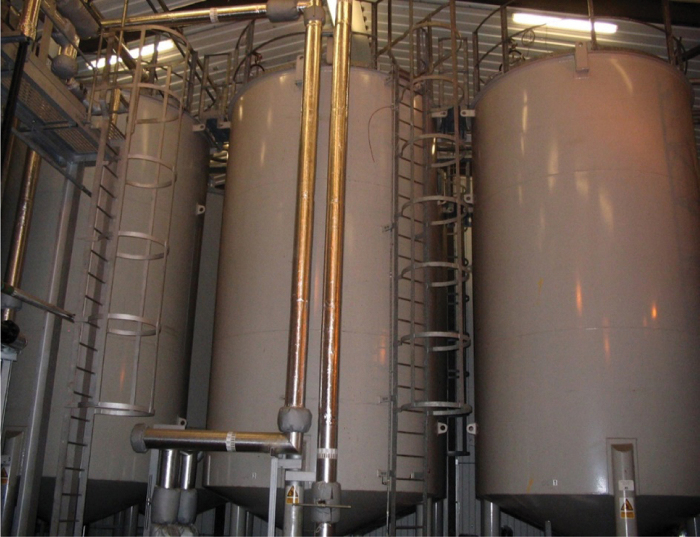
Effluent treatment plant storage vessels (before decontamination). Color images are available online.

**Figure 2.**
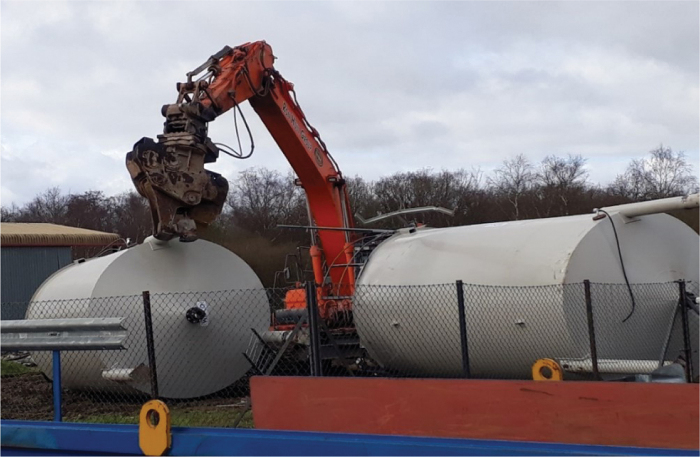
Removal of effluent treatment storage tanks after decontamination. Color images are available online.

For example, the system included six 20 m^3^ (approximately) storage vessels (Figures 1 and 2). It had not been normal practice to fill these vessels to 100% capacity when the laboratories were operational. In operations, they were rarely filled >20% (∼1 m head of pressure). This routine operation did not normally expose the discharge valve on the base of the vessel to the pressure a full fill with disinfectant would exert (i.e.,4–5 m head of pressure). To compound the situation, a 12% NaOH solution with a density of ∼1.13 kg/L would exert even greater pressure than the predominantly water-based effluent (density 1 kg/L) received while operational.

As such, given the caustic nature of NaOH, it was assessed too hazardous to fill these vessels to their maximal operation capacity. Instead, they were filled to their normal operating levels, and the remainder of the vessels internal walls were disinfected by manual spraying from access hatches. The kill tanks were considered decontaminated after their last treatment cycle of 95°C for 60 min. FMDV has been shown to be inactivated within 3 min at 67°C when in slurry form.^14^

An important consideration was to ensure no further effluent entered the kill tanks after their respective final validated kill cycles. The spent NaOH was pumped out of the system and bulk stored onsite before removal by a specialist contractor and not processed through the kill tanks, the usual route of flow when treating effluent. There were two important considerations here; first, the system was not designed to boil NaOH, a very hazardous chemical even at ambient temperatures and second, releasing such large volumes of a very alkaline liquid could be in breach of the sewage discharge license limits.

As thermal treatment vessels, the kill tanks were clad in insulation. The external cladding, which consisted of riveted steel sheets, was surface disinfected as part of the space, then removed to expose the insulation. The insulation was assessed to be negligible risk as it was enclosed by the cladding thereby protecting it from external contamination, and during routine treatment cycles the insulation was routinely exposed to high temperatures. In the unlikely event of an undetected leak from a kill vessel, any contents would have been repeatedly exposed to high temperatures (∼95°C at the point of contact with the vessel wall) resulting in rapid thermal inactivation. The insulation was then removed, bagged, and sent for incineration.

### Furniture, paperwork, and miscellaneous combustible materials

Material that could be readily incinerated was surface disinfected and where possible, placed in sealable burn bins, which were surface disinfected and securely stored onsite before incineration.

## Waste Management

A key element to the process was the management of waste, and more specifically using disposal routes that ultimately ensured waste was either destroyed, or otherwise rendered such that it was not reusable. Although at this stage all materials had been suitably decontaminated using a combination of time and disinfection processes, it was considered prudent to ensure waste products could not be reused offsite or identified as coming from the site, thereby mitigating reputational risk. Options available included incineration, smelting, and burial in a deep landfill. In addition, rubble from the building demolitions has been stored onsite leaving a cleared clean site for future development.

## Assurance

### Waste disposal sites

Assurance was provided by confirming the terminal waste sites, whether incinerators, landfill sites or others, were open to visits by the client. For example, both incinerators used to destroy waste from the site were visited by both biosafety and environmental representatives to give assurance of the process and to ask any questions.

### Risk assessment and documentation

Key to the whole process was the assessment of risk to reach a workable process accounting for time-related reduction in infectivity, practical methods of disinfection, and disposal of waste material. In addition, each building or part of building had a Decontamination Certificate signed off at stages by the responsible parties.

## Summary

The limitation of older buildings that could not be safely or effectively sealed for fumigation were overcome by the application of multiple risk reduction processes, including time and repeated disinfection with multiple disinfectants with different modes of action. In addition, waste materials were destroyed postdecontamination by incineration or smelting wherever possible to prevent reuse. The approach and methods described in this article were assessed as appropriate for the unique set of circumstances presented here (including the viruses worked on and the time buildings remained inactive before demolition) and are intended to give the reader some ideas on which to develop their own risk-based decontamination plan. In addition, some of the solutions and approaches developed were subsequently applied to decontaminate another building onsite as part of repurposing for lower containment work.
